# Development and intrapuparial characterization of *Peckia* (*Euboettcheria*) *collusor* (Curran and Walley, 1934) (Diptera: Sarcophagidae) for application in forensic entomology

**DOI:** 10.1038/s41598-024-65070-9

**Published:** 2024-07-09

**Authors:** Rayane Dias, Thais Viana, Jacenir R. Santos-Mallet, Jeronimo Alencar, Margareth M. C. Queiroz

**Affiliations:** 1 Laboratório Diptera, Instituto Oswaldo Cruz (Fiocruz), Avenida Brasil 4365, Manguinhos, Rio de Janeiro, RJ 21040-360 Brasil; 2 Programa de Pós-graduação em Biologia Animal, Universidade Federal Rural do Rio de Janeiro, Km 07, Zona Rural, BR-465, Seropédica, Rio de Janeiro 23890-000 Brazil; 3 Laboratório Integrado: Simulídeos e Oncocercose & Entomologia Médica e Forense (LSOEMF), Instituto Oswaldo Cruz - Fundação Oswaldo Cruz (IOC/FIOCRUZ), Pavilhão Herman Lent – Avenida Brasil, 4365, Manguinhos, RJ 21040-360 Brazil; 4 Programa de Pós-Graduação em Biodiversidade e Saúde, Instituto Oswaldo Cruz - Fundação Oswaldo Cruz (IOC/FIOCRUZ), Pavilhão Arthur Neiva – Avenida Brasil, 4365, Manguinhos, RJ 21040-360 Brasil; 5 Laboratório Interdisciplinar em Vigilância Entomológica em Diptera e Hemiptera, Instituto Oswaldo Cruz (FIOCRUZ), Av. Brasil, 4365, Manguinhos, RJ 21040-900 Brazil; 6 Universidade Iguaçu (UNIG), Av. Abílio Augusto Távora, 2134 – Luz, Nova Iguaçu, Rio de Janeiro 26260-045 Brazil

**Keywords:** Biology, Fly, Pupa, Immature, Post-mortem interval, Developmental biology, Drug discovery

## Abstract

The family Sarcophagidae is very diverse in Brazil. Due to their living habits, they are the subject of many medical, veterinary, sanitary, and entomological studies. However, Sarcophagidae species are still poorly studied in forensic entomology, although they are frequently reported in carcasses and even human corpses. Thus, this study aims to identify and compare the developmental stages and intrapuparial morphological characteristics of *Peckia (Euboettcheria) collusor* to serve as an auxiliary tool in forensic entomology. The pupae collected after zero hour at 27 °C and 32 °C were sacrificed every three hours until the first 24 h and then every six hours until the emergence of the first adults, using 30 pupae each time, totaling 1560 for 27 °C and 1290 for 32 °C. The intrapuparial development time of this fly species under laboratory-controlled conditions was 288 h at 27 °C and 228 h at 32 °C. The 2820 pupae were analyzed according to temperature and classified into eight possible stages. This contributed to the selection of 16 key morphological characteristics to identify the age of the pupae. The identified intrapupal morphological characteristics have great potential to help researchers, experts, technical assistants, and forensic entomologists estimate the minimum post-mortem interval (minPMI) of cadavers.

## Introduction

Some species of the order Diptera have a strong synanthropic association with humans. Among them, flies may present various levels of synanthropy according to the environmental conditions of a particular site, biotic factors like reproductive potential, eating habits, and competition, and abiotic factors like temperature and humidity^1^.

Flies are one of the first insect groups to arrive in corpses^2–4^, and the families Sarcophagidae and Calliphoridae are considered the main ones responsible for carcass colonization. This characteristic highlights the need for more in-depth biological studies on species development as a tool for forensic entomology, aiming to assist in crime investigation through information from the development cycle of these insects^5^. Adults of Sarcophagidae vary in size from small to large, have grayish or brown bodies, present three longitudinally blackened bands in their mesonotum, and their abdomen is characteristically checkered with spots varying in silver and gray tones^6^. The genus *Peckia* is a Sarcophagidae member with 67 identified species; one of them is *Peckia* (*Euboettcheria*) *collusor* (Curran and Walley, 1934), with records in South American countries such as Argentina, Bolivia, and Brazil^7^. In Brazil, studies on this species mainly focus on the bionomic aspects and post-embryonic development of immature forms^8,9^. *Peckia* (*Euboettcheria*) *colusor* is a necrophagous species whose larvae have a great affinity for decomposing organic matter. This characteristic makes it an interesting tool when associated with cadavers for forensic entomology, which directly depends on studies focusing on taxonomy, biology, life cycle, succession, and the ecology of cadaveric fauna^10^. In this sense, those with necrophagous habits are the most used to estimate the minimum post-mortem interval (_min_PMI) through the estimated age of the immature forms^11^.

In this context, the current study aimed to identify and compare the developmental stages and intrapuparial morphological characteristics of *Peckia* (*Euboettcheria*) *collusor*, with the goal of using the generated data as an auxiliary tool in forensic entomology (Supplementary Information).

## Results

**Figure 1:**
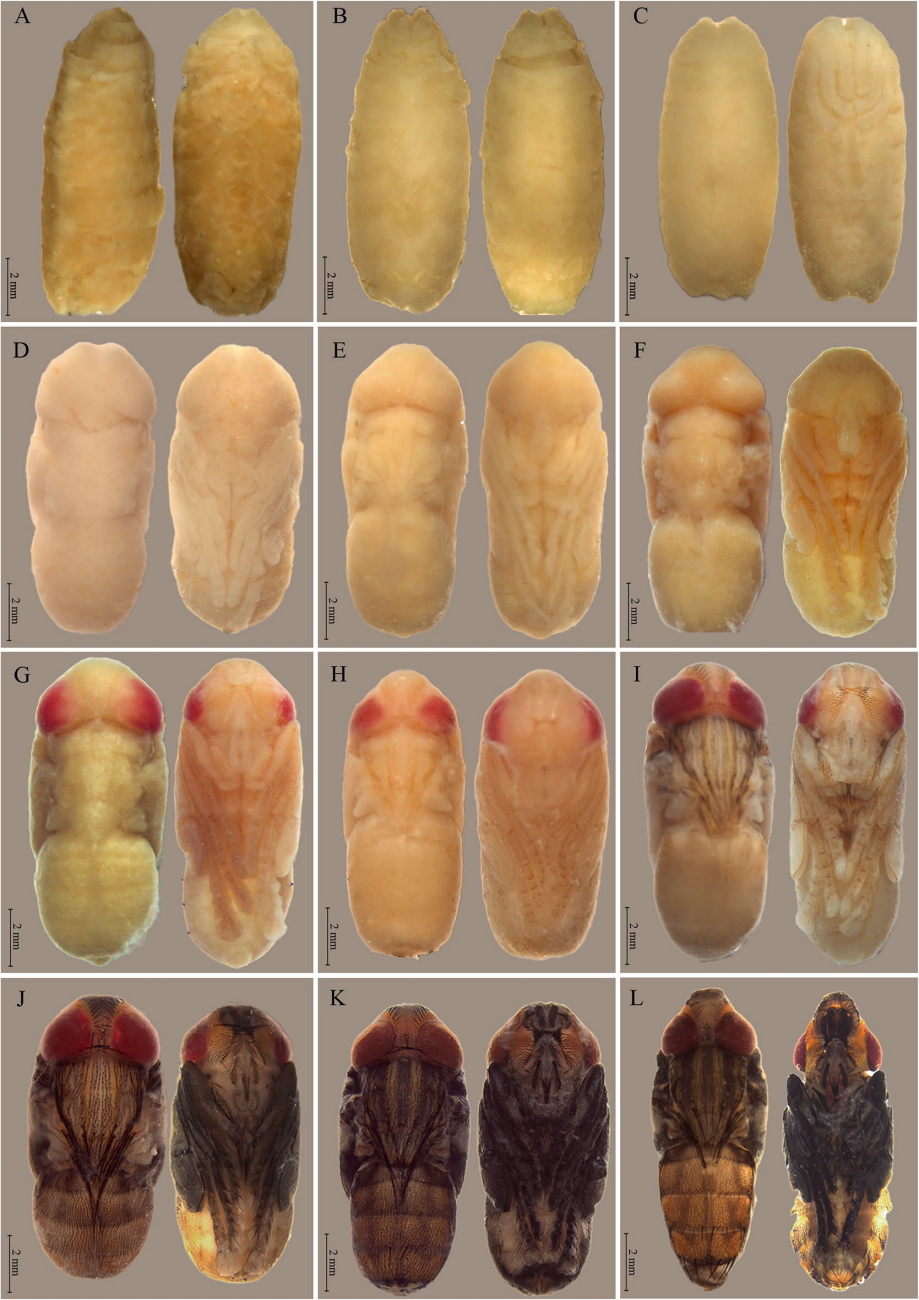
Stages of intrapuparial development of *Peckia* (*Euboettcheria*) *collusor* at 27 °C: (**A**) pre-pupa at 9 h; (**B**) anterior cryptocephalic pupa in dorsal and ventral view (emergence of the legs) at 36 h; (**C**) posterior cryptocephalic pupa in dorsal and ventral view (thoracic appendages reaching 1/4 of the body) at 54 h; (**D**) phanerocephalic pupa in dorsal and ventral view (larger wings and legs) at 102 h; (**E**) pupa-adult apolysis in dorsal and ventral view (head structures being formed) at 120 h; (**F**) adult pharate with yellow eyes and whitish wings at 168 h; (**G**) adult pharate with more defined and uniform eyes with pink coloration at 174 h; (**H**) adult pharate with formation of head structures such as antennae and buccal apparatus (maxillary palps) at 188 h; (**I**) adult pharate with the appearance of the bristles in the cephalic and thoracic region, in addition to the darkening of the costal vein of the wing and definition of the three bands in the thorax at 204 h; (**J**) adult pharate with red eyes, with arista in the antennae and darker veins and legs at 198 h; (**K**) adult pharate indicating fully pigmented structures at 276 h; (**L**) imago with expanded ptilineal suture at 288 h. Scale: 2 mm.

**Figure 2:**
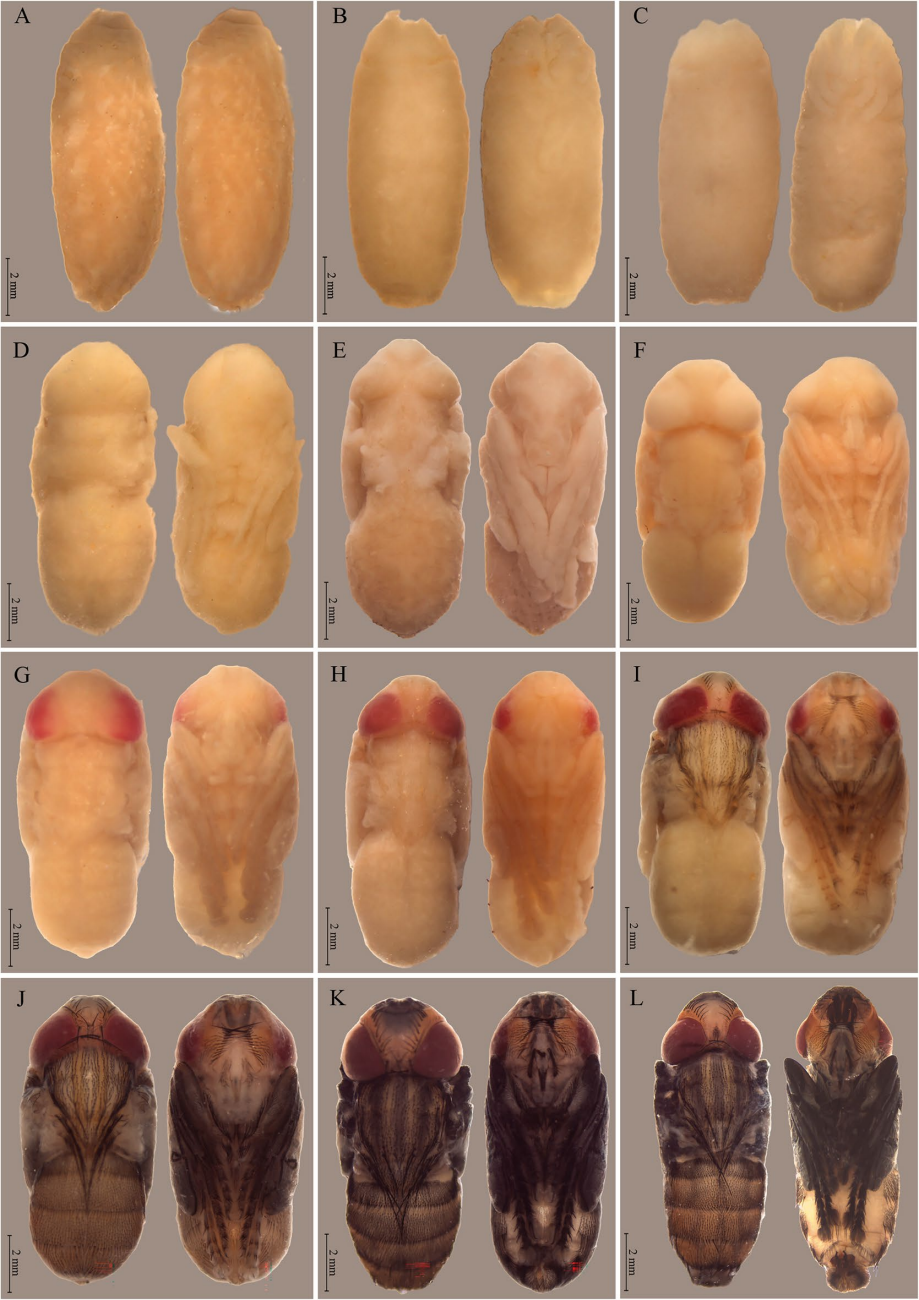
Stages of intrapuparial development of *Peckia* (*Euboettcheria*) *collusor* at 32 °C: (**A**) pre-pupa at 12 h; (**B**) anterior cryptochephalic pupa in dorsal and ventral view (small legs) at 30 h; (**C**) posterior cryptochephalic pupa in dorsal and ventral view (legs reaching 1/4 of the body) at 60 h; (**D**) phanerocephalic pupa in dorsal and ventral view (wings and legs gaining definition) at 126 h; (**E**) pupa-adult apolysis in dorsal and ventral view (the structures of the head beginning to take shape) at 144 h; (**F**) adult pharate with yellow eyes and whitish wings at 156 h; (**G**) adult pharate with pink colored eyes at 168 h; (**H**) adult pharate with emergence of antennae and buccal apparatus at 180 h; (**I**) adult pharate with the emergence of bristles and definition of the three bands in the thorax at 186 h; (**J**) adult pharate with red eyes, with arista in the antennae and veins at 192 h; (**K**) adult pharate indicating fully pigmented structures at 210 h; (**L**) Imago with expanded ptilineal suture and well-formed genital apparatus at 228 h. Scale: 2 mm.

Data were obtained by observing 2820 pupae, 1560 at 27 °C and 1260 at 32 °C. Morphological structures were recorded from their onset until the emergence of adults of *P.* (*E*.) *collusor* at 27 °C, which occurred after 288 h, equivalent to 12 days (Fig. 1), and at 32 °C, which occurred after 228 h, equivalent to 9.5 days (Fig. 2).

Puparia of* P* (*E*.) *collusor* measured approximately 7.65 mm in length and 3.15 mm in width. On the surface of the puparia, from 0 to 12 h, it is possible to see the larval segmentation, and throughout the development, its coloration attains brown tones that remain throughout its development.

The developmental stages were divided into eight stages. After characterizing the interval of each developmental stage, it is possible to identify at what point morphological structures arise that can assist in estimating the age of the immature (Table 1), thus conferring a better _min_PMI through the detailed data.

**Table 1:** Minimum and maximum time of appearance of the main morphological characteristics for estimating the age of the *Peckia* (*Euboettcheria*) *collusor* pupa at 27 and 32 °C under laboratory conditions.

Morphological markers of pupas	27° C		32 °C	
	Min	Max	Min	Max
Emergence of the legs	15	48	15	36
Thoracic appendages (legs and wings) reaching 1/4 of the body	30	54	24	48
Evaginated head	42	*	30	*
Yellow eyes	168	198	132	168
Onset of the definition of antennae, buccal apparatus, and legs	126	156	102	144
Pink eyes	168	222	138	180
Onset of wing innervation	192	264	162	198
Emergence of bristles (legs, head, and buccal apparatus)	186	*	138	*
Darkening of legs	192	270	168	258
Red eyes	192	*	180	*
Transparent ocelli	186	*	162	*
Presence of arista in the antenna	198	*	168	*
Pigmented antennae	210	*	198	*
Reddish maxillary palps	210	*	174	*
Three bands on the thorax	234	*	180	*
Genital apparatus formed (males ≠ females)	228	*	198	*

*Emergence time interval in hours.

**Figure 3:**
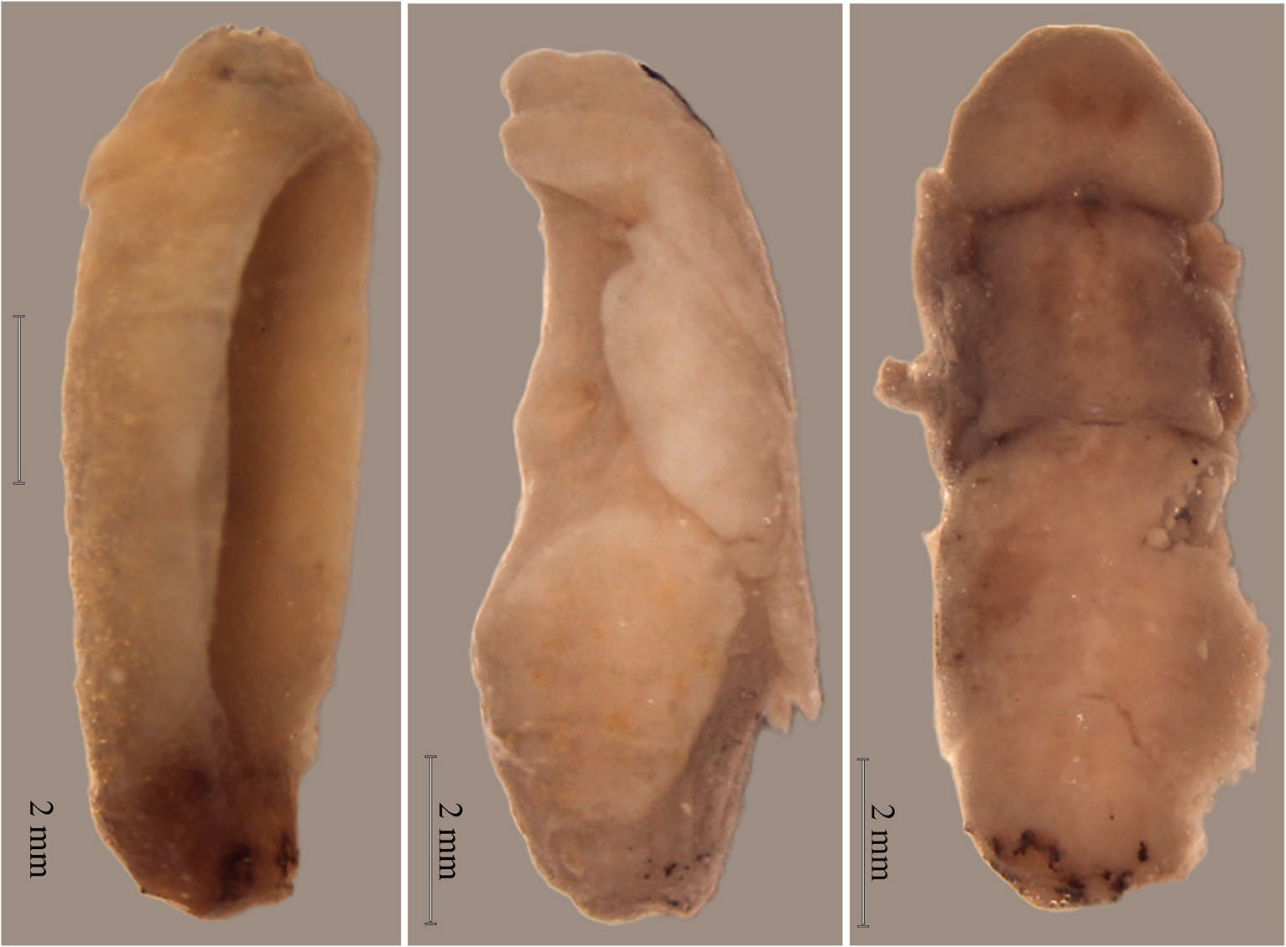
Abnormalities present in dead pupae.

Some specimens presented abnormalities such as spots and deformations that were recorded at both temperatures, with a higher percentage at 32 °C, equivalent to a mortality rate of 2.9% (Fig. 3).

The pupae that did not present abnormalities were used for the detailed analysis of the characteristics (Tables 2 and 3) corresponding to the developmental stages according to each stipulated temperature.

**Table 2:** Sample number (N), minimum and maximum values (development interval), and time (mean and standard deviation, in hours) of each stage of intrapuparial development of *Peckia* (*Euboettcheria*) *collusor* at 27 °C, under laboratory conditions.

Stages	N	IV (Range)	X ± (SD)
Pupariation	30	0	
Pre-pupae	186	3–36	11.65 ± 5.46
Early cryptococephalic pupae	108	15–48	27.11 ± 6.73
Late cryptococephalic pupae	26	30–54	39.46 ± 5.57
Phanerocephalic pupae	282	42–162	77.66 ± 21.29
Pupa-adult apolysis	304	72–168	128.17 ± 20.45
Adult pharate			
Yellow eyes	49	168–198	175.84 ± 6.81
Pink eyes	122	168–222	189.05 ± 10.97
Red eyes	424	192–288	241.70 ± 22.09
Adult (Emergence)	2	288	0.00
Dead/puparium	29		

N = number of pupae per stage.

IV = Range of variation (hours).

X ± SD = mean ± standard deviation (hours).

**Table 3:** Sample number (N), minimum and maximum values (development interval), and time (mean and standard deviation, in hours) of each stage of intrapuparial development of *Peckia* (*Euboettcheria*) *collusor* at 32 °C, under laboratory conditions.

Stages	N	IV (Range)	X ± (SD)
Pupariation	30	0	
Pre-pupae	178	3–36	11.26 ± 5.32
Early cryptococephalic pupae	72	15–42	23.00 ± 5.14
Late cryptococephalic pupae	115	24–66	44.97 ± 11.09
Phanerocephalic pupae	278	30–138	79.06 ± 17.52
Pupa-adult apolysis	214	108–168	133.29 ± 14.16
Pharate			
Yellow eyes	29	132–168	148.34 ± 9.57
Pink eyes	88	138–180	167.93 ± 6.91
Red eyes	219	180–228	201.83 ± 11.72
Adult (Emergence)	3	228	0.00
Dead/puparium	37		

N = number of pupae per stage.

IV = Range of Variation (hours).

X ± SD = Mean ± Standard Deviation (hours).

The characteristics of the intrapuparial development of *Peckia* (*Euboettcheria*) *collusor* described below were observed at 27 ± 1 °C.

### Pupariation (0 h)

The larvae collected during this period were classified based on their feeding cessation, locomotion interruption, and subsequent head inversion, making removing the puparium difficult.

### Pre-pupae (3–36 h)

The dissected pupa presents marks resulting from the strong and recent association with the puparium. It has an off-white coloration delimited by a tangle of cells with no defined characteristics.

### Early cryptocephalic pupae (15–48 h)

In this period, the imaginal discs begin to give rise to rudimentary thoracic appendages at the apex of the pupa, where previously, the cephalopharyngeal skeleton was present. The appendages in this period do not exceed one-fourth of the total body size of the pupa, and the head remains invaginated.

### Late cryptococephalic pupae (30–54 h)

This period is marked by thoracic appendages still without definition and/or segmentation, occupying more than one-fourth of the total size of the pupal body. This is a short stage in which the head remains invaginated, and the rest of the body remains whitish without defined characteristics.

### Phanerocephalic pupae (42–162 h)

The pupa corresponding to this period presents a completely everted head. The thoracic appendages have already appeared to their full extent but have not yet begun their differentiation processes. The phanerocephalic pupa has rudimentary eyes and mouthparts. From 66 h, the anterior spiracles are positioned lateral-anteriorly, exhibiting a yellowish coloration.

### Pupa-adult apolysis (72–168 h)

During this period, the differentiation of thoracic appendages becomes evident, and it is already possible to clearly differentiate legs and wings that gradually acquire specific characteristics, culminating in the segmentation of the legs that begins at 108 h, later giving rise to the thigh, trochanter, femur, tibia, tarsus, and post-tarsus. The buccal apparatus is more evident, and the palps appear as small protuberances, still very immature. This period is also marked by the beginning of bristle development in the gena, legs, wings, and dorsal region of the thorax. The abdomen still does not present defined characteristics, nor do the eyes, which are still rudimentary.

### Pharate adult (168–282 h)

This stage is characterized by the beginning of eye differentiation, with yellowish pigmentation at the beginning, between 168 and 198 h, followed by pinkish pigmentation between 168 and 222 h, and culminating in red pigmentation, which begins at 192 h, until adult emergence (Fig. 2). At 186 h, it is possible to observe prominent antennae and ocular triangle, as well as bristles in the ocular triangle, between eyes, antenna, legs, and palps. The darkening of bristles on the legs and thorax marks the 192-h interval. At 198 h, the bristles become evident at the base of the wings, pronotum, abdomen, and rostrum. The antenna is yellowish, and bands are observed in the dorsal view of the thorax, which is also yellowish. At 204 h, there is a slight yellowing in the last abdominal tergite, forehead, parafacialia, parafrontalia, and gena, and the bristles originated in the thorax reach the third tergite of the abdomen. Darkening of the bristles of the rostrum and buccal apparatus is also observed. At 210 h, the buccal apparatus can be differentiated by the reddish coloration of the labrum-labella, and the antennae present brown pigmentation. The onset of genitalia differentiation defines the period of 216 h. At 228 h, the darkening of wing veins and maturation of the sexual apparatus occurs, evidencing differences between males and females in relation to the terminalia. At 234 h, the thorax presents dark bands between the yellowish ones previously identified. A strong orange pigmentation in the last tergite of the abdominal portion is also observed. At 270 h, there is a total darkening of the antennae, legs, buccal apparatus, palps, and wings; at 282 h, adults are already fully formed, waiting only for the moment of emergence.

### Imago (288 h)

In this period, the emergence of adults begins as a consequence of the end of intrapuparial development.

## Discussion

In Brazil, *Peckia* sp. is highly abundant in carcasses^12^. Its immature stages show good development in organic matter at different decomposition states^4,13^. The duration of the pupal stage of some species has already been studied for this genus; however, for *P.* (*E.*) *collusor*, a species of medical-veterinary and forensic importance, this had not yet been described, despite being a necrophagous species with applicability in forensic entomology^4,14–16^ and recorded in South America in countries such as Argentina, Bolivia, Brazil, among others^17,18^.

**Figure 4:**
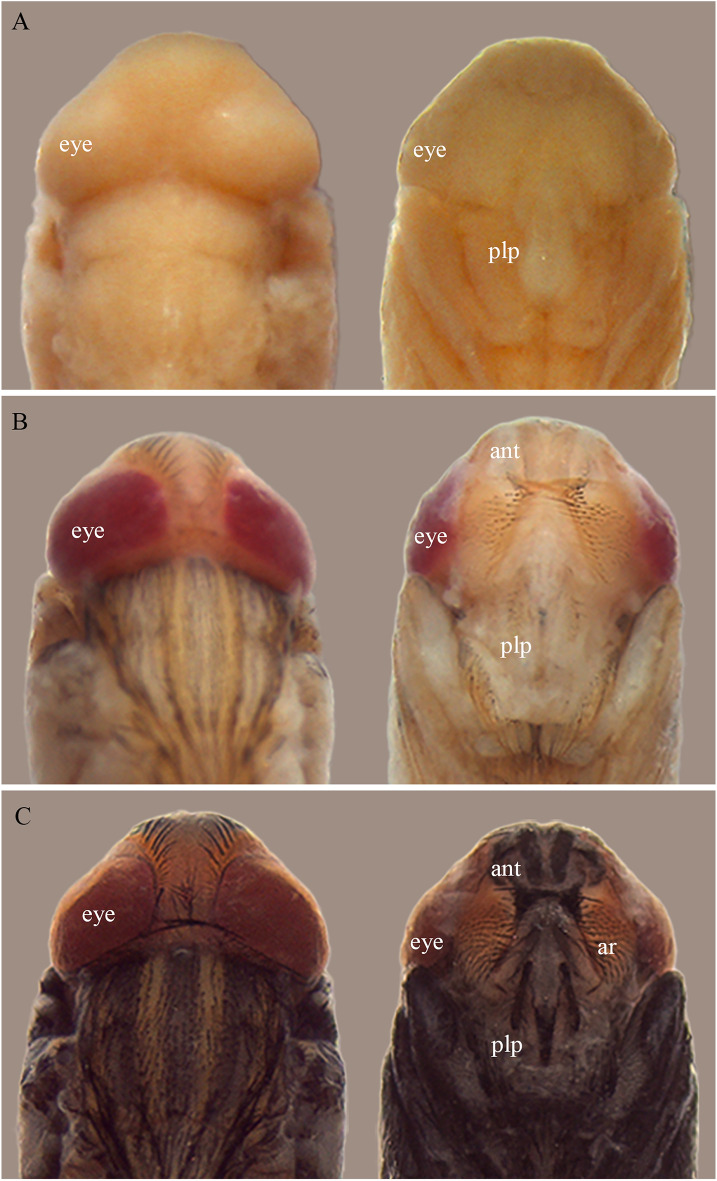
*Peckia* (*Euboettcheria*) *collusor* in dorsal and ventral view of head development in relation to the coloration of the compound eyes of the pharate at 27 °C: yellow eyes at 168 h (**A**); pink eyes at 204 h (**B**); red eyes at 276 h (**C**). Abbreviations: ant, antenna; plp, maxillary palpus; ar, arista. Scale: 2 mm.

**Figure 5:**
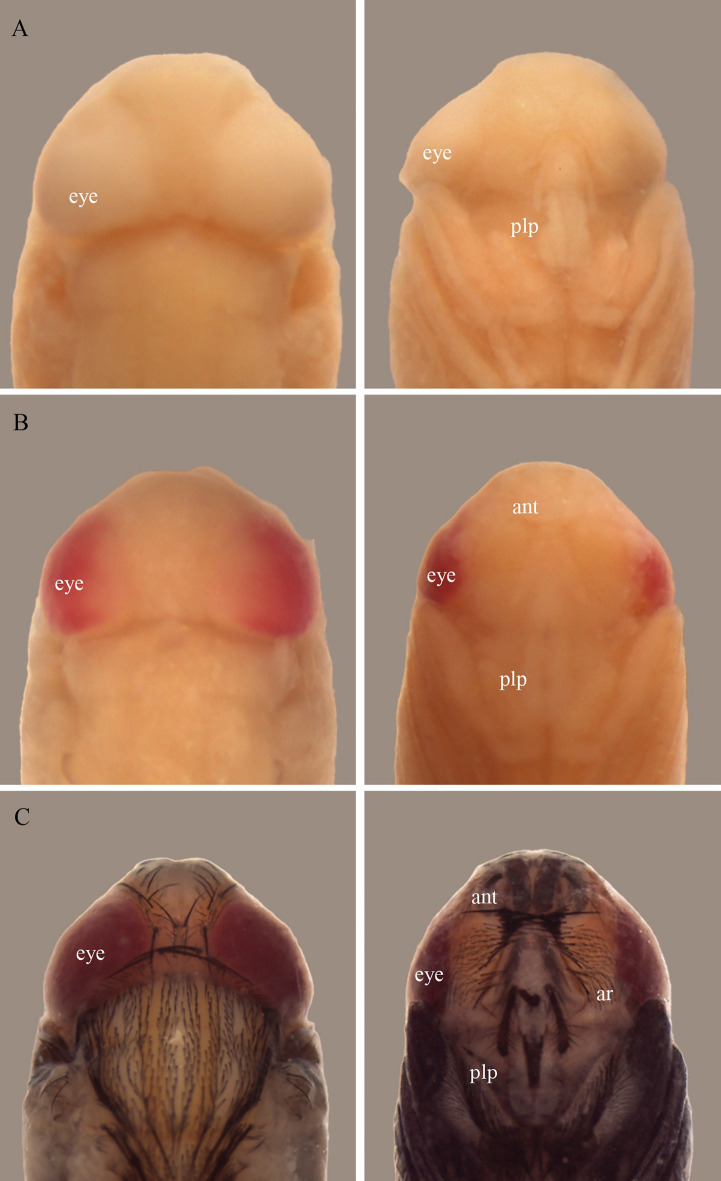
*Peckia* (*Euboettcheria*) *collusor* in dorsal and ventral view of head development in relation to the coloration of the compound eyes of the pharate at 32 °C: well-defined yellow eyes at 156 h (**A**); pink eyes at 168 h (**B**); red eyes at 210 h (**C**). Abbreviations: ant, antenna; plp, maxillary palpus; ar, arista. Scale: 2 mm.

The pupal period was chosen as it represents the majority of the immature development stage^19^ and consequently has the greatest number of characteristics due to the metamorphosis that occurs in this phase, leading to the emergence of adult traits. Moreover, it is a phase without locomotion and feeding. Therefore, it does not require as much care as the other life cycle stages: once found in a location, there is no need to wait for its application, thus contributing to a more accurate estimate of the _min_PMI. During observations of the pupal period, the pharate adult stage was the longest at both temperatures, presenting many changes, emphasized mainly by the pigmentation of the eyes. This characteristic determines this stage (Figs. 4 and 5). It is imperative to have a parameter indicating the beginning and end of each stage, as well as a pattern that can be followed when classifying a given stage^20^.

Describing morphological changes during intrapuparial development under laboratory conditions is necessary because this period varies according to temperature, humidity, and photoperiod^21^. The pupal stage is characterized by the onset of the major modifications that will characterize an adult insect; therefore, determining the moment of appearance of each modification through study of the post-embryonic and intrapuparial development of specimens in the laboratory contributes to PMI calculation^21–25^. Therefore, studies like ours seek to identify and compare the developmental stages and intrapuparial morphological characteristics to serve as an auxiliary tool in forensic entomology.

The development time of dipterans, especially muscoids, may differ due to external factors. Among abiotic factors, temperature strongly influences the development of sarcophagids and other species of forensic importance^26,27^. The present results evidence this influence since the increase or decrease in temperature delayed or accelerated the development. Therefore, similar analyses carried out at different temperatures are necessary to enable the use of data in different localities and regions, as well as in the case of the body being found in places that cause an increase or decrease in temperature, such as car bags, buried or in a closed environment, allowing a closer analysis of the environment of the crime scene and offering greater specificity of the development of the immature forms present there.

Cunha^28^ conducted a similar experiment with *Peckia* (*Pattonella*) *intermutans* (Walker, 1861) and *Peckia* (*Sarcodexia) lambens* (Wiedemann, 1830) under controlled conditions regarding temperature, photoperiod, and humidity. The imagos of *P.* (*P.*) *intermutans* emerged at 318 h at 23 °C of development, those of *P.* (*S.*) *lambens* at 234 h at 21 °C, 126 h at 26 °C, and 114 h at 31 °C. In the present study, the imagos of *P.* (*E.*) *collusor* displayed similar behavior, with faster development at the highest temperature analyzed, as well as the pharate adult stage, which corresponded to most of the intrapuparial period. However, the total development time differed between species of the same genus^28^, highlighting the importance of analyzing each species separately. In the present study, *P.* (*E.*) *collusor* had a shorter development time in relation to these species, as the imagos emerged in 288 h at 27 °C and 228 h at 32 °C. *Peckia* (*Peckia*) *chrysostoma* (Wiedemann, 1830) showed a pupal development time of 23.5 days at 18 °C and 12.5 days at 27 °C, and *Peckia* (*Squamatodes*) *ingens* (Walker, 1849) of 33 days at 18 °C and 16 days at 27 °C, differing in this regard from other congeneric species^29^.

*Peckia* (*Sarcodexia*) *lambens* resulted in larval viability of 97.5% at 26 °C and 93% at 31 °C, while pupal viability was 98% and 87%, respectively^28^. Thus, in the current study, it is also possible to assume that a temperature close to 25 °C is best for these species through the mortality rate of 1.9% at 27 °C and 2.9% at 32 °C for *P.* (*E.*) *collusor*. The same is true for the optimum humidity, whose ideal range for insect survival is between 40 and 80% R.H.^30^. Based on this, a 60% R.H. was determined.

Nascimento et al.^31^ conducted a detailed study of the bionomy of *P.* (*E.*) *collusor*, considering its developmental stages at a temperature of 27 °C, which was also used in the current study and to describe the stages of intrapuparial development. Their results corroborate those described in the present study since at 27 °C, it has an average duration of 12 days, close to what Nascimento et al.^31^ obtained, which was 11.3 ± 9 days. Pupal viability, when compared, shows that the temperature of 27 °C is the most favorable for its development in both studies, which is practically above 75%. This similarity demonstrates that the life cycle of *P.* (*E.*) *collusor* in this condition will present responses close to this time range. The comparison between studies on bionomics or development provides greater veracity to the data of the present study for its use as a tool to assist in estimating the PMI.

Shang et al.^32^ used different methodologies to estimate the pupal age of *Sarcophaga peregrina* (Robineau–Desvoidy, 1830), a sarcophagid of forensic importance. The intrapuparial development of this species was divided into 12 substages, six associated with external morphology using light microscopy and 10 associated as well with internal morphology of the pupa by histological analysis. The authors found this analysis to be an important complementary method to determine stages of intrapuparial development. These same authors also used gene expression and found that six genes (*NDUFS2*, *CPAMD8*, *NDUFV2*, *Hsp27*, *Hsp23*, and *TPP*) can be used to estimate the pupal age of *S. peregrina*. The emergence of adults was approximately 1,090.3 ± 30.6 h at 15 °C, 566.6 ± 21.9 h at 20 °C, 404.6 ± 13.01 h at 25 °C, and 280.3 ± 4.5 h at 30 °C.

Duarte et al.^33^ conducted a study on *Sarcophaga* (*Liopygia*) *ruficornis* (Fabricius, 1794) at temperatures of 22 °C, 27 °C, and 32 °C. They observed the emergence of adults at 440 h, 272 h, and 232 h, respectively. Compared to the present results, *P*. (*E*.) *collusor* exhibited a longer development time at both temperatures, with emergence at 288 h at 27 °C and 228 h at 32 °C.

Duarte and Queiroz^34^ studied the intrapuparial development of *Hydrotaea aenescens* (Wiedemann, 1830), a species from the family Muscidae, which is also of forensic importance. Their methodology was very similar to the present study and that conducted by Couto and Queiroz^35^ on another species from the same family, *Musca domestica* (Linnaeus, 1758). Both species displayed shorter life cycles at all temperatures compared to *P.* (*E*.) *collusor*. The differences were also significant across the stages, notably in the adult emergence phase, where *Hydrotaea aenescens* occurred at 174 h at 27 °C and 126 h at 32 °C, while *Musca domestica* emerged at 138–144 h at 27 °C and 96–112 h at 30 °C.

The studies found in the literature on intrapuparial development are mostly about the Calliphoridae species^36–39^. The reported species were *Hemilucilia semidiaphana*, *Sarconesia chlorogaster* (Wiedemann, 1830), *Chrysomya putoria* (Wiedemann, 1818), *Calliphora vicina*, (Robineau-Desvoidy, 1830), *Cochliomyia macellaria* (Fabricius, 1775), *Lucilia illustris* (Meigen, 1826), all with shorter development times.In addition, insights into their reproductive biology, accurate identification, and rearing aspects are instrumental in facilitating forensic studies. However, it is vital to identify species with longer pupal stages, such as those in the family Sarcophagidae, for use in situations where a corpse is found several days post-mortem, as species with shorter development periods would have likely completed their emergence.

## Conclusion

The morphological characteristics of *P.* (*E.*) *collusor* presented here can serve as a solid basis for future studies of Sarcophagidae species, which are commonly found in forensic cases. The analysis of the 16 key characteristics found during the pupal stage, pointing to the moment of emergence, shape, and coloration in detail, as well as the eight stages described, contribute to the development of tools that can help in forensic cases through a practical and low-cost method for its applicability in forensic laboratories. Photographic records of the main changes that occur in each stage facilitate the work of professionals from different research areas, experts, technical assistants, and forensic entomologists in the sense that they can reliably apply this information in studies and cases. The body or scene does not always provide enough information to solve the case. Therefore, our study contributes to interdisciplinarity between the areas that are increasingly common and necessary for high-quality studies.

## Material and methods

### Specimen collection and colony establishment

*Peckia* (*E.*) *collusor* adults were collected at the Fundação Oswaldo Cruz (FIOCRUZ) campus in Manguinhos (22° 52′ 29.64′′ S; 43° 14′ 43.39′′ W), in the city of Rio de Janeiro, Brazil, in a secondary succession forest area of the Atlantic Forest, in March 2020. An active collection was carried out using a modified Shannon trap containing pork meat as bait, with 24 h of exposure at room temperature. After this period, the adults were captured using Falcon tubes.

The collected specimens were transported to the Integrated Laboratory: Simulidae and Oncocerciasis & Medical and Forensic Entomology (*Simulídeos e Oncocercose & Entomologia Médica e Forense*—LSOEMF/FIOCRUZ) where the adults were identified according to the key of Vairo et al.^40^. After identification, they were placed in wooden cages (30 × 30 × 30 cm), packed in a ventilated shelf at a temperature of 27 ± 1 °C, with a 12-h photoperiod and relative humidity (RH) of 60 ± 10%.

### Obtention of larvae

Female larviposition was stimulated through the supply of putrefied ground beef on a Petri dish for 48 h. The larvae obtained were transferred to plastic containers containing 2 g of decomposed ground beef/larva. This container was placed into a larger one containing sufficient vermiculite (inert soil) as a substrate to cover its entire base, enabling the pupariation of third-instar larvae. All containers were stored under photoperiod conditions of 12 h (L:D) and RH of 60 ± 10%. A B.O.D. germination chamber was used for the temperature control of 32 ± 1 °C, and a ventilated shelf was used for the temperature control of 27 °C^41^.

### Intrapuparial development study

A minimum sample number was calculated for each temperature based on pupal duration and biology of *P.* (*E.*) *collusor*.

In the first 24 h, specimens were collected at the time of pupariation (characterized by head retraction) every three hours and separated into tubes with groups of 10 specimens per replicate. For each time interval observed, three replicates were conducted. The tubes were sealed with hydrophobic cotton and labeled with the corresponding date and time. They were then exposed to high-temperature water (~ 75 ± 5 °C) to euthanize the pupae inside. After the first 24 h, pupae were collected every six hours until the emergence of the first adult. Thirty pupae were collected for each interval (Supplementary Information).

The puparia with the dead pupae inside were fixed in Carnoy’s solution for 48 h, then transferred to tubes containing 5% formic acid for another 48 h. At the end of this period, they were stored in Eppendorf tubes and frozen until dissection, which was performed with forceps and hypodermic needles. The dissected pupae were immersed in 70% ethanol in Eppendorf tubes duly registered according to their collection times and temperatures and photographed under a Leica EZ4 HD^®^ Germany model stereoscopic microscope (4,4:1) recording of morphological changes relevant to determine the age of *P.* (*E.*) *collusor* and its developmental stages, based on Fraenkel and Bhaskaran^42^, Martín-Vega et al.^19^, and Zhang et al.^43^.

### Supplementary Information

Supplementary Information.

## Data Availability

The datasets used and/or analyzed during the current study are available from the corresponding author upon reasonable request.
